# Online Social anxiety Cognitive therapy for Adolescents (OSCA): protocol for a randomised controlled trial

**DOI:** 10.1186/s13063-019-3651-6

**Published:** 2019-10-07

**Authors:** Eleanor Leigh, David M. Clark

**Affiliations:** Department of Experimental Psychology, University of Oxford, OxCADAT, The Old Rectory, Paradise Square, Oxford, OX1 1TW UK

**Keywords:** Adolescence, Social anxiety disorder, Cognitive behaviour therapy, Internet therapy, Psychological therapy, Young people

## Abstract

**Background:**

Adolescent social anxiety disorder (SAD) is common, impairing and persistent. There is a need to intervene early to avert its long-term consequences. Cognitive Therapy for SAD is the leading treatment for adults and shows promise for adolescents. However, given the scale of the problem of adolescent SAD and the limited availability of psychological therapists in child and adolescent mental health services, there is a substantial gap in service provision. Delivering therapy via the Internet may provide part of the solution to this problem. An Internet version of adult Cognitive Therapy for SAD has been developed, with outcomes similar to face-to-face therapy. We have recently adapted this treatment for use with adolescents with SAD. Here, we describe a randomised controlled trial designed to test the efficacy of Internet Cognitive Therapy for adolescent SAD compared to waitlist.

**Methods/design:**

Forty adolescents aged 14–18 years with a diagnosis of SAD will be recruited via schools. Participants will be randomly allocated to Internet Cognitive Therapy or to waitlist. All participants will be assessed three times during the study—at baseline (pretreatment/wait), midtreatment/wait (week 8) and posttreatment/wait (week 15). Participants in the experimental arm will also complete weekly measures as part of the online program and they will be assessed at 3 and 6 months. Postwait, participants in the waitlist arm will be offered Internet Cognitive Therapy, and weekly and posttreatment data will also be collected for them. The trial aims to test whether Internet Cognitive Therapy is superior to waitlist in reducing social anxiety symptoms and in reducing the proportion of adolescents meeting criteria for SAD. Other outcomes of interest include depression and general anxiety symptoms. Acceptability of the online treatment will also be evaluated.

**Discussion:**

This randomised controlled trial will provide preliminary evidence on whether this intervention, requiring relatively low levels of therapist input, is safe and clinically effective. If this is shown to be the case, Internet Cognitive Therapy for adolescents has the potential to provide a service to the large population of adolescents with untreated SAD.

**Trial registration:**

ISRCTN Registry, ISRCTN15079139. Version 1 registered on 06/02/2019.

**Electronic supplementary material:**

The online version of this article (10.1186/s13063-019-3651-6) contains supplementary material, which is available to authorized users.

## Background

Social anxiety disorder (SAD) is the third most common mental health disorder [1], with prevalence rates estimated to be as high as 10% by the end of adolescence [2]. Almost all cases of SAD occur during the adolescent years [1]. It is associated with substantial impairment in relationships [3], educational attainment [4], and day-to-day functioning, as well as an elevated risk of further anxiety and depressive disorders [5]. For the majority of sufferers, the disorder is characterised by a persistent course [6, 7]. Intervening in the early stages of SAD has the potential to offset the multiple, negative consequences associated with the disorder.

Currently the most common psychological treatment approach for adolescents with SAD is generic forms of cognitive behaviour therapy (CBT) developed for a range of anxiety disorders. Unfortunately, young people with SAD have significantly poorer outcomes from these treatments compared to those with other anxiety disorders [8–13]. For example, for 7–17 year olds, remission from SAD was 40.6% compared to 72% for separation disorder or generalised anxiety disorder [9]. Disorder-specific psychological therapies (e.g. [14–16]) have been shown to outperform waitlist but there is limited evidence of specific treatment effects, in the sense of being superior to other credible interventions. In contrast, for *adults* with SAD, a form of individual psychological therapy, Cognitive Therapy based on the Clark and Wells [17] cognitive model, has been shown to be more effective than a wide range of alternative therapies in a network meta-analysis (Mayo-Wilson et al., [18]) and is one of the first-line treatments recommended by the National Institute for Health and Care Excellence (NICE, [19]). Turning to young people, the NICE guideline recommends that the use of Cognitive Therapy should be considered for older adolescents with SAD. In line with this, we have demonstrated promising findings in a case series (Leigh & Clark, [20]) in which we adapted Cognitive Therapy for adolescents. Furthermore, Ingul et al.([21] undertook a randomised controlled trial (RCT) comparing individual therapy based on the Clark and Wells cognitive model [17] to group CBT [22] and an attention placebo. Even though the individual therapy provided in the trial was an attenuated form of Cognitive Therapy for SAD, it was found to outperform both comparator conditions, providing preliminary evidence of specific treatment effects. Taken together, it seems that Cognitive Therapy based on the Clark and Wells model may hold promise as an effective treatment for adolescent SAD [23].

Whilst there are encouraging advances in treatment development for adolescent SAD, there are major obstacles to treatment access and service provision. Most young people with mental health disorders do not receive any treatment and the treatment gap is particularly large for youth with anxiety disorders [24]. For example, the US National Comorbidity Survey reported that less than one in five adolescents received services for an anxiety disorder, and only 12% of young people with SAD received any treatment, rising to 21% amongst those with severe SAD [24]. Even for those who do access treatment, the majority do not receive an adequate, evidence-based intervention [25]. There are many reasons for the unmet need. These include parent and child level factors, such as stigma, lack of awareness and low trust in professionals [26], and also service level factors, including failure to detect SAD by primary care professionals, limited resources of Child and Adolescent Mental Health Services (CAMHS), leading to long waiting times, and a limited number of trained therapists [27].

There is clearly a need to develop effective, scalable treatments in order to bridge the treatment gap that exists for the majority of adolescents with SAD. One potential solution to the problem of limited access is the use of the Internet to deliver therapy. Potential benefits to services of Internet-delivered therapy include reduced therapist time and reduced burden on clinics, for example, by lowering the need for clinic space. There are also a number of potential advantages of Internet therapy to young service users and their families. Internet therapy may be more convenient because young people can use the treatment programme when it suits them. Travel time and costs for attending sessions are minimised. The disruption to schooling caused by therapy sessions, typically scheduled during working hours, is also almost eliminated. Young people interact with the online environment in all aspects of their lives and so receiving treatment this way may be especially appealing to this cohort. Accessing a treatment online may provide a greater sense of confidentiality and reduced embarrassment [28] which will be especially important for adolescents with social anxiety for whom feelings of shame and embarrassment are typical [29].

Our team have developed and tested an Internet version of Cognitive Therapy for SAD for *adults*. Therapist contact is all remote (email, text, phone) and therapist time is reduced to only 20% of that in face-to-face therapy [30]. A recent RCT with socially anxious adults found that the Internet therapy has similar effects to face-to-face Cognitive Therapy (Clark et al., in preparation). We have recently undertaken a consultation study with young people to identify how this adult Internet treatment needs to be adapted to meet the needs of an adolescent SAD population. Only minor adaptations were suggested, for example ensuring that case examples are relevant to young people (e.g. describing adolescent-relevant situations, such as being at school and seeing friends, rather than being at work).

The primary aim of this Online Social anxiety Cognitive therapy for Adolescents (OSCA) trial is to examine the effectiveness of Internet Cognitive Therapy for adolescents with SAD compared to waitlist within a school setting.

We hypothesise that treatment with OSCA will be superior to waitlist in reducing symptoms of social anxiety and that the proportion of adolescents meeting criteria for social anxiety disorder will be lower posttreatment compared to postwait. We hypothesise that gains will be maintained at 3- and 6-month follow-up. We also expect that treatment with OSCA will be superior to waitlist in reducing symptoms of general anxiety and depression. We will gather data on the acceptability of the treatment. We will also examine whether proposed psychological process variables mediate any observed change in social anxiety symptoms after OSCA, and whether we can predict who will benefit in particular from OSCA. The results of the present clinical trial will be used to improve access to effective psychological treatments for socially anxious adolescents.

## Methods/design

### Design

**Fig. 1:**
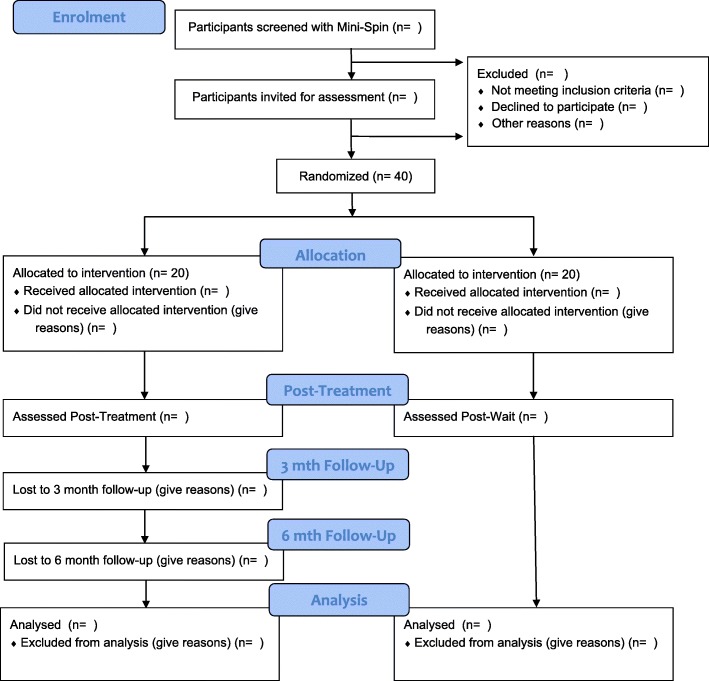
OSCA trial flowchart

The study is a two-arm RCT conducted in secondary schools in the United Kingdom (UK). The experimental arm is Online Social anxiety Cognitive therapy for Adolescents (OSCA). The comparison arm is waitlist. All participants will be assessed three times: at baseline (pretreatment/wait), midtreatment/wait (week 8) and posttreatment/wait (week 15). Participants in the experimental arm will also be assessed at 3- and 6-month follow-up. Postwait, participants in the waitlist arm will be offered OSCA. Assessments will be undertaken with parents as well where possible. Figure 1 shows the trial flow diagram. The trial has received approval from the University of Oxford Medical Sciences Division Research Board and it has been prospectively registered (http://www.isrctn.com/ISRCTN15079139).

**Fig. 2:**
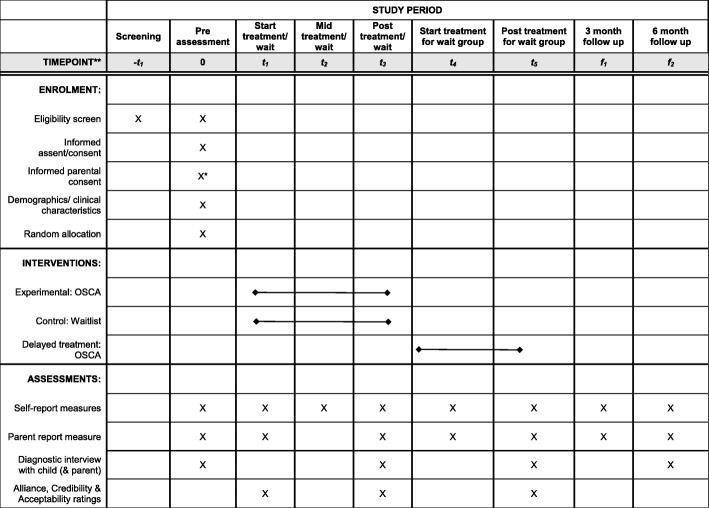
SPIRIT figure for OSCA trial

The basic trial methods of enrolment, interventions, and assessments are summarised in Fig. 2. The Standard Protocol Items: Recommendations for Interventional Trials (SPIRIT) checklist is provided as Additional file 1.

### Participants

A total of 40 adolescents aged between 14 and 18 years at intake with a diagnosis of SAD will be included. Diagnostic and Statistical Manual of Mental Disorders, 5th edition (DSM-5) diagnosis will be determined by the Anxiety Disorders Interview Schedule for children and parents [31]. Only adolescents for whom SAD is the primary problem will be included. All participants must be able to read and write in English due to the nature of the therapy and the need to complete self-report questionnaires.

Young people who are currently receiving any other psychological intervention or who have previously received Cognitive Therapy or CBT for SAD will not be invited to take part. Other exclusion criteria include: the presence of an autism spectrum disorder; learning disability; current alcohol or substance dependence; presence or suspected presence of psychosis; suicidal intent or recurrent self-harming behaviour; and young people identified by social services as currently ‘at risk’ due to child protection concerns.

### Sample size

Sample size was estimated from an RCT of adolescents with SAD comparing a version of face-to-face Cognitive Therapy with attention placebo (Ingul, Aune, & Nordahl, 2014). The comparison effect size posttreatment was 1.26. A recent adult RCT by our group suggests outcomes for face-to-face and Internet-delivered Cognitive Therapy are similar. Based on a conservative estimate of the effect of 1.0, we would need 17 per group for 80% power. The sample size was inflated to allow for drop out following randomisation [32], resulting in a final sample size of 40.

### Recruitment and randomisation

Participants will be recruited via secondary schools. The use of schools as a referral source has two advantages. It will ensure adolescents with a range of symptom severity will be included and it fits with potential future delivery mechanisms for the treatment, should efficacy be demonstrated.

For the screening stage, during class time in school, young people will be asked to complete the Mini-SPIN [33] and to indicate whether or not they would like to learn more about the study. The Mini-SPIN is a three-item questionnaire that is sensitive and specific to SAD in adults [34] and adolescents [35]. We were guided in how best to recruit participants in a non-stigmatising way in consultation with school students. Prior to initiating recruitment in schools we will send information out to parents informing them of the project and providing details of what will be happening in their child’s school.

The process of seeking informed consent will differ for 14 to 15 year olds and 16 to 18 year olds. This is because 16 to 18 year olds will be treated as ‘competent youth’ for the purposes of this study. For 14 to 15 year olds, information about the study will be provided to the young person after parental consent has been obtained. For 16–18 year olds, information about the study will be provided to the young person directly, without seeking parental consent first. At least 24 h after information has been given to the young person, a meeting will be scheduled during school hours. At this meeting written assent/consent will be sought. Once written assent/consent has been obtained, the full pre-intervention assessment will be completed. This will involve ensuring all eligibility criteria are met. For 16–18 year olds, we will also seek their agreement to contact parents in order to inform them of their child’s involvement in the project.

Adolescents meeting inclusion criteria and not meeting any exclusion criteria will be randomised to waitlist or treatment. Adolescents not eligible to take part after the parent and young person assessments will be supported in accessing help via alternative means (if they would like this). Eligible participants will be randomly allocated to OSCA or waitlist. Individual randomisation will be conducted using an online minimisation algorithm generated by the trial statistician. The ratio will be 1:1. Minimisation will ensure balance between trial arms for gender, but will retain a random element and will be stratified by severity. Randomisation will occur after consent has been taken and baseline measures have been completed. It will be done by the Oxford Cognitive Health and Neuroscience Clinical Trials Unit. The trial team will email details of the stratification variables to the independent statistician who will then randomise the participant. Following randomisation, participants will be notified of their allocation.

### Interventions

OSCA uses the same Internet platform as the adult programme with minor adaptations. We undertook a small study in which five young people (aged 16–18 years) worked through the adult online programme and then provided their feedback and suggestions for adaptations for adolescents. The programme was well received, and only minor adaptations were suggested. Specifically, the young people suggested that some video clips be re-filmed with young actors and that the case examples were relevant to young people (e.g. describing adolescent-relevant situations, such as being at school and seeing friends, rather than being at work).

The OSCA program takes 14 weeks. All users receive a core set of modules to work through at the beginning of the programme. The programme is then individualised for each user. The therapist releases modules that will be most helpful to that person, depending on their particular concerns. Adolescents complete OSCA modules at home and they can logon as often as they like. During the 14 weeks of treatment, young people allocated to OSCA will have a 15-min phone conversation with their therapist each week, in line with procedures in the adult treatment trial (Clark et al., in preparation). The telephone call is to support and encourage young people and to ensure that they are given access to all of the parts of the program that are most helpful to them. In addition, they will receive regular encouragement and support via secure messaging within the online programme and SMS texts.

We will seek to keep all parents updated on their child’s progress in treatment. This will be explained to children aged 14–15 years. Consent for this will be sought from young people aged 16–18 years. Parental involvement will involve regular emails with a short general summary of therapy modules.

The only contact with participants in the waitlist arm during the wait period will be at the midwait time point, when they are requested to complete outcome measures. Participants in the waitlist arm will be offered treatment with OSCA after the postwait assessment.

One of the benefits of Internet treatments is their high fidelity [36]. The therapist (EL) was involved in the development of Cognitive Therapy for SAD in adolescents. Throughout the trial they will receive supervision from the developer of the cognitive model of SAD, the face-to-face and Internet versions of Cognitive Therapy for SAD, and the version for adolescents (DMC).

### Outcomes

#### Primary outcome measures

The primary outcomes will be changes on the self-report version of the Liebowitz Social Anxiety Scale for Children and Adolescents–Self-report Version (LSAS-CA-SR) [37], and recovery from SAD. To assess recovery, the proportion of adolescents who continue to meet DSM-5 diagnostic criteria for SAD at posttreatment/wait will be examined. Diagnosis will be made using The Anxiety Disorders Interview Schedule IV for Children and Parents (ADIS-C/P) [31]. All participants will be interviewed with the child version and parents will be interviewed where possible. Participants who withdraw from the study will also be invited to complete the assessments. Assessments will be completed face-to-face or over the telephone by trained assessors. See Table 1 for timings of outcome measures.

**Table 1:**
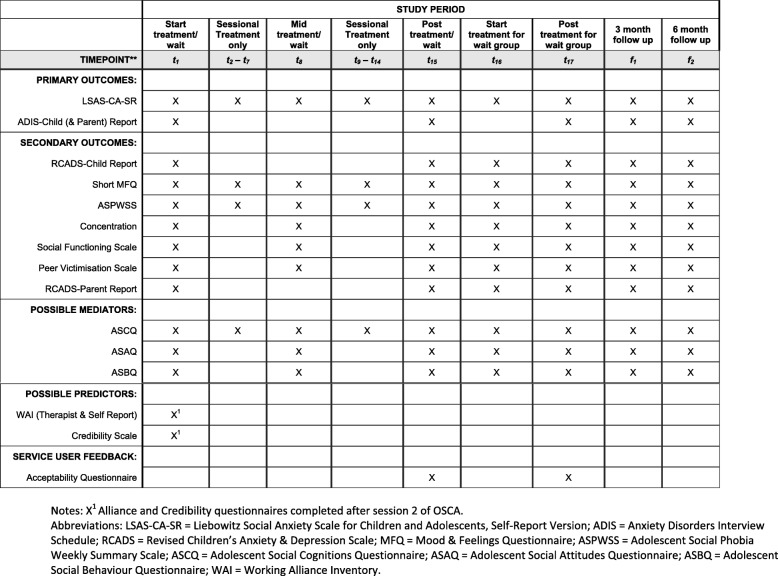
Schedule of self-, parent- and therapist-report measures

#### Secondary outcome measures

As secondary outcomes, changes on a measure of aspects of social anxiety (the Social Phobia Weekly Summary Scale [38]), general anxiety as measured by the Revised Child Anxiety & Depression Scale (RCADS, self-report [and parent report where possible]) [39] and depression as measured with the Short Mood and Feelings Questionnaire (SMFQ) [40] will be examined. Social functioning (including social satisfaction, friendship quality and peer victimization) will be assessed by self-report questionnaire and school functioning will be captured through percentage attendance and grade average (either internal or external examination scores or grade estimate). Self-reported ability to concentrate in class will be measured [20]. See Table 1 for timings of outcome measures.

The following measures of possible mediators of therapeutic improvement will be used: the Adolescent Social Cognitions Questionnaire (ASCQ); Adolescent Social Behaviours Questionnaire (ASBQ); and Adolescent Social Attitudes Questionnaire (ASAQ). These three measures were all adapted for adolescents by the authors from versions developed for socially anxious adults [38].

#### Measure of alliance and treatment credibility and acceptability

The quality of the therapeutic relationship, as perceived by both the therapist and the participants, will be assessed with the shortened Working Alliance Inventory [41] as a potential predictor of outcome. Participants will rate treatment credibility with the Credibility of Therapy Scale [42] as another potential predictor of outcome. Feedback questionnaires will be completed posttreatment to assess the acceptability of the treatment.

### Blinding

Blinding of therapists and participants is not possible due to the nature of the design. However, assessments will be carried out by independent raters who are blind to allocation. The independent raters will be psychology graduates or above. All will have received training in the use of the outcome assessment. All will have demonstrated reliability in administration of the ADIS-C/P.

### Data completeness and monitoring

In our adult trials of Cognitive Therapy, data completeness has always been above 95%. Methods to enhance data completeness that we will adopt include the use of session-by-session outcome monitoring (so if someone drops out early the last available symptom measure can be used) and weekly outcome-measure informed supervision. As a phase I trial, a Data Management Committee was not considered to be necessary. As such, the trial team is responsible for monitoring and management of the data. Data will be monitored for completeness, consistency and plausibility by the trial statistician. The trial team and statistician will have full access to the final trial dataset. The study data will be reported in line with current CONSORT guidelines.

### Statistical analysis

All analyses will be intent-to-treat. No interim analyses are planned. Outcomes will be compared with hierarchical linear modelling. Time (midwait/midtreatment and postwait/posttreatment), treatment condition (OSCA, waitlist) and the time–condition interaction will be specified as categorical fixed factors, with baseline LSAS and gender as fixed covariates and participant as a random effect to account for between-person variation. Exploratory analyses using linear mixed effects models for each step to account for the nested data structure [43] will test for mediation of OSCA on social anxiety symptoms at posttreatment through candidate process variables from the cognitive model. Baseline outcomes will be included as predictors in all models. Categorical outcomes (response, remission, deterioration and diagnostic status) will be analysed using logistic regression, with treatment condition as the independent variable and baseline LSAS score and gender as covariates.

### Safety aspects

Potential risks to the participant are minimal. There is potential for inconvenience because the young person is invited to take part in a treatment that will involve a time commitment. The potential for inconvenience will be minimised by virtue of the online nature of the treatment: young people can decide when and where are most convenient for them to complete treatment modules. Young people are free to withdraw from the study at any time.

In principle, there is potential for distress while completing OSCA. The likelihood of this is low because the program is goal-oriented in nature; young people did not become upset receiving the treatment in our previous case series. We will minimise this risk by fully explaining the nature of the program to young people. The young person is free to withdraw from the project at any time. In our pilot case series none of the adolescents who were treated showed an overall deterioration in their symptoms. Instead, everyone showed some benefit. This is consistent with the findings from our adult trials. We therefore consider the risk of symptom exacerbation to be low. Weekly measures of social anxiety and depressed mood will be taken and so any signs of deterioration will be detected quickly in the unlikely event that this should occur. An item assessing risk to self will be inserted into the SMFQ from the long version of the scale (item 19). Scores on this item will be reviewed weekly by the therapist to monitor levels and changes in risk. If signs of deterioration or an elevation in risk are identified, then the young person will be contacted and the appropriate procedures will be followed. An aim of the OSCA trial is to examine the safety of the treatment. We will do this in two ways. Adverse events (any untoward occurrence in an individual to whom the intervention has been administered, including occurrences which are not necessarily caused by or related to that therapy) will be monitored and recorded from randomisation to the final follow-up at 6 months. We will also assess reliable deterioration on the LSAS-CA-SR (our primary outcome measure).

Half of the participants will be randomly allocated to the waitlist condition. During this 14-week period the only contact they will have with the research team is at the midwait stage when they will be asked to complete questionnaires. It is possible that participants in the waitlist condition will require treatment during this period. Participants and their parents will be advised that they should seek services and help as required.

The online treatment programme has numerous security features representing current best practice and complies with NHS data security standards. It employs secure client-server communication, full encryption of the server database, enforcement of strong passwords, two- factor authentication and hosting on a tier 4 hosting server. External access to the database using SSH protocol is prohibited. The system has been subjected to industry-standard penetration testing. Online data are secured by encryption to prevent access from outside parties. Access to the server data by the software company hosting the online therapy programmes (FRY-IT) is protected by non-disclosure agreements and the Data Protection Act (1998).

### Service user involvement

Young people, both healthy school pupils and service users, have been involved in all stages of the project. Service users have been involved in the development of the questionnaires and the worksheets used to support face-to-face Cognitive Therapy and OSCA. Young people have provided detailed advice on how to adapt adult online Cognitive Therapy for young people. Pupils have advised on how best to recruit young people in schools. A small group of young people have requested to remain involved in a consultation role throughout the trial.

## Discussion

Given the prevalence of social anxiety in adolescents and the very limited number of child therapists in the NHS, we are faced with the challenge of developing effective, scalable treatments. Here, we describe the protocol for a RCT that aims to examine the efficacy of a newly developed online version of Cognitive Therapy for SAD in adolescents (OSCA) compared to waitlist control. If superiority of OSCA can be demonstrated, this will have important implications for treatment provision.

The majority of adolescents with SAD are not accessing services despite experiencing high levels of symptoms and impairment [24]. There may be a number of reasons for this, including perceived stigma and shame, a lack of understanding of the care system or a shortage of appropriate CAMHS services. Two aspects of the OSCA trial design have the potential to overcome at least some of these obstacles and improve outcomes for socially anxious young people.

Using the Internet to delivery therapy provides a number of practical advantages over face-to-face therapy, including reduced therapist and clinic burden and high treatment fidelity. In addition to this, as highlighted in the recent Government Green Paper (2017), schools may provide an ideal setting to intervene early in youth mental health difficulties, potentially overcoming various obstacles to accessing care. For example, it would sidestep the potential issue of underdetection of SAD by primary care physicians [27]. Likewise, screening programs are likely to identify young people with SAD who have not sought help, for example due to feelings of shame, embarrassment or failure by family members to appreciate the severity of the problem. It would also reduce the burden and interference associated with attending appointments at a clinic.

## Conclusions

The aim of the trial is to determine whether OSCA, an Internet version of Cognitive Therapy for adolescent SAD delivered via schools, is effective compared to a waitlist control. Data about the acceptability of the treatment and suggestions for ways to improve OSCA will be sought from young people and parents. If the intervention is found to be effective compared to waitlist, then a future trial could compare the Internet intervention to face-to-face therapy, to examine clinical outcomes and costs. OSCA has the potential to improve the treatment outcomes and future prospects for adolescents with SAD, the majority of whom are not currently receiving treatment, as well as reduce the burden on over-stretched CAMHS services.

## Additional file

Additional file 1: SPIRIT 2013 Checklist: Recommended items to address in a clinical trial protocol and related documents. (DOC 119 kb)

## Data Availability

After publication of the study results, the individual participant data will be made available via the Bodleian-ORA data repository. Data available will be research data reported in the publication, with the exception of data that could compromise participant anonymity.

## Abbreviations

ADIS-C/P: Anxiety Disorders Interview Schedule for Children and Parents
ASAQ: Adolescent Social Attitudes Questionnaire
ASBQ: Adolescent Social Behaviour Questionnaire
ASCQ: Adolescent Social Cognitions Questionnaire
CAMHS: Child and Adolescent Mental Health Service
CBT: Cognitive behaviour therapy
CONSORT: Consolidated Standards of Reporting Trials
DSM-5: Diagnostic and Statistical Manual of Mental Disorders, 5th edition
LSAS-CA-SR: Liebowitz Social Anxiety Scale for children and adolescents self-report version
Mini-SPIN: Mini Social Phobi Inventory
NICE: The National Institute for Health and Care Exellence
OSCA: Online Social anxiety Cognitive therapy for Adolescents
RCADS: Revised Children’s Anxiety and Depression Scale
RCT: Randomised controlled trial
SAD: Social anxiety disorder
SMFQ: Short Mood and Feelings Questionnaire
SPIRIT: Standard Protocol Items: Recommendations for Interventional Trials
WAI: Working Alliance Inventory
